# Effects of Natural Products on Fructose-Induced Nonalcoholic Fatty Liver Disease (NAFLD)

**DOI:** 10.3390/nu9020096

**Published:** 2017-01-31

**Authors:** Qian Chen, Tingting Wang, Jian Li, Sijian Wang, Feng Qiu, Haiyang Yu, Yi Zhang, Tao Wang

**Affiliations:** 1 Tianjin Key Laboratory of TCM Chemistry and Analysis, Institute of Traditional Chinese Medicine, Tianjin University of Traditional Chinese Medicine, 312 Anshan Road, Nankai District, Tianjin 300193, China; serafinachen@163.com (Q.C.); 18202682964@163.com (T.W.); yuhaiyang19830116@hotmail.com (H.Y.); 2 Tianjin State Key Laboratory of Modern Chinese Medicine, 312 Anshanxi Road, Nankai District, Tianjin 300193, China; beyondwill@126.com (J.L.); 15122587883@163.com (S.W.); fengqiu20070118@163.com (F.Q.)

**Keywords:** fructose-induced NAFLD, lipogenesis, natural products, mitochondrial dysfunction, inflammatory pathways, insulin resistance

## Abstract

As a sugar additive, fructose is widely used in processed foods and beverages. Excessive fructose consumption can cause hepatic steatosis and dyslipidemia, leading to the development of metabolic syndrome. Recent research revealed that fructose-induced nonalcoholic fatty liver disease (NAFLD) is related to several pathological processes, including: (1) augmenting lipogenesis; (2) leading to mitochondrial dysfunction; (3) stimulating the activation of inflammatory pathways; and (4) causing insulin resistance. Cellular signaling research indicated that partial factors play significant roles in fructose-induced NAFLD, involving liver X receptor (LXR)α, sterol regulatory element binding protein (SREBP)-1/1c, acetyl-CoA carboxylase (ACC), fatty acid synthase (FAS), stearoyl-CoA desaturase (SCD), peroxisome proliferator–activated receptor α (PPARα), leptin nuclear factor-erythroid 2-related factor 2 (Nrf2), nuclear factor kappa B (NF-κB), tumor necrosis factor α (TNF-α), c-Jun amino terminal kinase (JNK), phosphatidylinositol 3-kinase (PI3K) and adenosine 5′-monophosphate (AMP)-activated protein kinase (AMPK). Until now, a series of natural products have been reported as regulators of NAFLD in vivo and in vitro. This paper reviews the natural products (e.g., curcumin, resveratrol, and (−)-epicatechin) and their mechanisms of ameliorating fructose-induced NAFLD over the past years. Although, as lead compounds, natural products usually have fewer activities compared with synthesized compounds, it will shed light on studies aiming to discover new drugs for NAFLD.

## 1. Introduction

Fructose—also known as fruit sugar—is a ketonic monosaccharide present in many plants, such as sugar cane, sugar beets, and corn. It has been appreciated for many years that fructose is applied as a sugar additive, typically in high fructose corn syrup, the main ingredient of soft drinks, pastries, desserts, and other daily processed foods. It is postulated that large quantities of added sugars entering the daily diet will enhance the possibility of suffering from nonalcoholic fatty liver disease (NAFLD) [1,2]. Randomized clinical trials revealed that a reduction of sugar-sweetened beverage intake for 6 to 24 months significantly reduced individual body weight gain [3]. Exploratory observational pilot studies showed that over-consumption of fructose in NAFLD patients may increase risk of liver steatosis [4], up-regulate circulating adropin concentrations [5] and lipocalin 2 level [6,7], increase hepatic fibrosis [8], and finally lead to cirrhosis and liver failure.

Many of clinical trials and ecological studies have assessed the close relationship between excessive consumption of fructose and NAFLD. It has been reviewed that overeating fructose may be the “caries” at the epidemic’s root [9]. The majority of the metabolic disorder of fructose is related to its rapid utilization through the liver, leading to increasing lipogenesis and very low density lipoprotein (VLDL) secretion, which finally causes various liver dyslipidemia [10]. Fructose enters hepatocytes through a transporter, especially glucose transporter 2 (Glut2), Glut5, Glut8 [11], or possibly Glut9 [12], where it is preferentially metabolized by fructokinase to generate fructose-1-phophate, then serves as a relatively unregulated source providing carbon atoms for both the glycerol and the acyl portions of triglycerides [13]. Consequently, fructose is a highly efficient inducer of de novo lipogenesis, which has been shown to reduce hepatic insulin sensitivity and increase the formation of VLDL [14]. The major clinical diseases based on hepatic dyslipidemia include obesity, NAFLD, and multiple metabolic syndromes, among which the prevalence of NAFLD has become a global problem in recent decades. It is reported that about 30% of the population in Western countries has been affected with NAFLD, and up to 15% among the overseas Chinese community [15]. Patients with NAFLD tend to suffer from non-alcoholic steatohepatitis, hepatic fibrosis, cirrhosis, hepatoma, and metabolic syndrome, meaning that it is a leading health risk in this population [16,17]. The incidence of metabolic syndrome distributes widely around the world, and the number of people with this syndrome has risen in epidemic proportions during the last 50 years [18]. It is necessary to certify clinical recommendations that fructose intake be controlled by limiting the consumption of sugar (especially high fructose containing)-sweetened foods or drinks. With little effective medicines discovered, there are a certain number of natural products reported to show bioactivity in depressing fructose-induced NAFLD. This paper reviewed the bioactive natural products on NAFLD recently reported in the literature, which can serve as a lead for new drugs research and diary supplement development.

## 2. Mechanisms of Fructose Action in NAFLD

Fructose is principally metabolized by fructokinase to generate fructose-1-phophate, irregularly providing glycerol phosphate and acyl coenzyme A, resulting in triglyceride formation that is both secreted and stored in hepatocytes [12,13]. Excessive fructose intakes adversely impact hepatic lipid metabolism and insulin sensitivity. Over-loaded fructose increases hepatic metabolic burden, stimulating the overproduction of acetyl-CoA in mitochondria [10]. Through tricarboxylate transport system, acetyl-CoA enters the cytoplasm to be used for fatty acid and cholesterol synthesis by regulation of liver X receptor (LXR)α, sterol regulatory element binding protein (SREBP)-1/1c, acetyl-CoA carboxylase (ACC), fatty acid synthase (FAS), and stearoyl-CoA desaturase (SCD).

**Figure 1:**
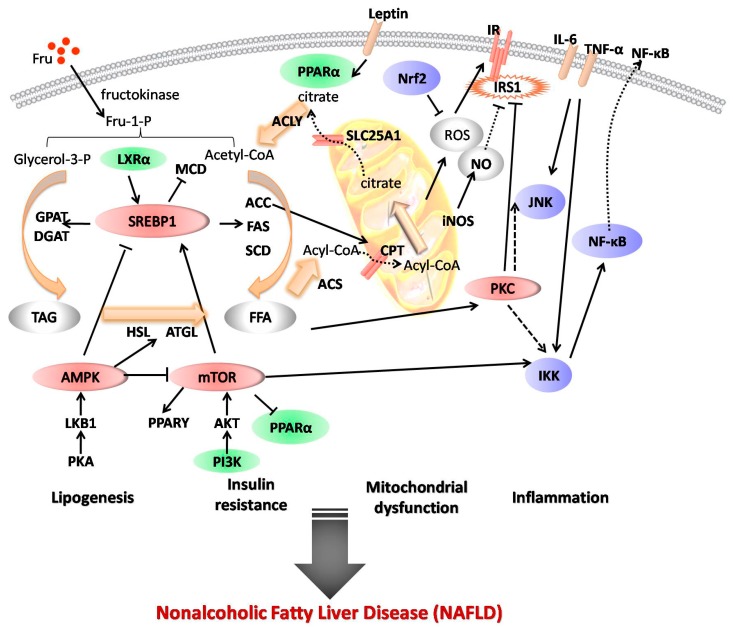
Schematic representation of underlying mechanisms of fructose-induced NAFLD. Fructose may stimulate NAFLD through different pathological processes, including: (1) augmenting lipogenesis through up-regulating liver X receptor (LXR)α and sterol regulatory element binding protein (SREBP)-1c; (2) leading to mitochondrial dysfunction by depressing peroxisome proliferator-activated receptor α (PPARα) and nuclear factor-erythroid 2-related factor 2 (Nrf2); (3) stimulating the activation of inflammatory pathways; and (4) causing insulin resistance. ACC: acetyl-CoA carboxylase; ACLY: ATP-citrate lyase; AKT: protein kinase B; AMPK: adenosine 5′-monophosphate (AMP)-activated protein kinase; ATGL: adipose triglyceride lipase; CPT: carnitine palmitoyl transferase; DGAT: diacylglycerol acyltransferase; FAS: fatty acid synthase; Fru: fructose; FFA: free fatty acid; GPAT: glycerol-3-phosphate acyltransferase; HSL: hormone-sensitive lipase; IKK: inhibitor of nuclear factor κB (IκB) kinase; IL-6: interleukin 6; iNOS: inducible nitric oxide synthase; IRS1: insulin receptor substrate-1; JNK: c-Jun amino terminal kinase; LKB1: Liver kinase B1; MCD: malonyl-CoA decarboxylase; mTOR: mammalian target of rapamycin NF-κB: nuclear factor kappa B; NO: nitric oxide; PI3K: phosphoinositide 3-kinase; PKA/C: Protein kinase A/C; ROS: reactive oxygen species; SCD: stearoyl-CoA desaturase; TAG: triacylglycerol; TNF-α: tumor necrosis factor α.

Consequently, increasing lipogenesis in the liver leads to the intracellular accumulation of malonyl-CoA, which represents a balance between synthesis from acetyl-CoA by ACC and utilization in fatty acid synthesis by FAS, as well as degradation to acetyl-CoA via the action of malonyl-CoA decarboxylase. Finally, an excess of malonyl-CoA leads to abnormal production of leptin [19], inhibits hepatic lipid β-oxidation, and increases the formation of reactive oxygen species (ROS), causing mitochondrial dysfunction with down-regulation of peroxisome proliferator-activated receptor α (PPARα)—a major transcription factor involved in the regulation of fatty acid oxidation. Due to the inhibition of nuclear factor-erythroid 2-related factor 2 (Nrf2), antioxidant capability is attenuated, and oxidative stress is thus implacable. Furthermore, ROS triggers inflammatory pathways involving nuclear factor kappa B (NF-κB), tumor necrosis factor α (TNF-α), and c-Jun amino terminal kinase (JNK), leading to hepatic inflammation via the insulin receptor signaling pathway, and further decreases insulin sensitivity, resulting in hepatic insulin resistance [20]. High de novo lipogenesis and oxidative stress are also connected with a deficiency of insulin sensitivity through phosphoinositide 3-kinase (PI3K) and adenosine 5′-monophosphate (AMP)-activated protein kinase (AMPK) pathways [21,22,23] (Figure 1).

Solid lines with arrowheads indicate known signaling events and direct stimulatory modification between lipogenesis, mitochondrial dysfunction, inflammation, and insulin resistance. Solid lines with short beelines represent direct inhibitory modification. Dashed lines with arrowheads denote tentative or indirect stimulatory modification. Dashed lines with short beelines mean tentative or indirect inhibitory modification.

## 3. Effects of Natural Products on Fructose-Induced NAFLD

Many natural products are reported to show regulation effects on fructose-induced NAFLD. Several promising drugs based on studies in rodents have been reviewed against fructose-induced fatty liver and endotoxins influx from the intestine [24]. According to the pathogenesis of fructose-induced NAFLD, the relevant natural products can be classified by their different functions, including regulation of lipogenesis, repair of mitochondrial dysfunction, inhibition of inflammatory pathways, and improvement of insulin resistance.

### 3.1. Regulation of Lipogenesis

Over-consumption of fructose causes an excess of acetyl-CoA in the mitochondria entering the cytoplasm to be used for fatty acid, leading to over-lipogenesis in the liver. Many natural products show a regulation effect on fructose-induced lipogenesis.

*Symplocos cochinchinensis*—a popular Indian herbal medicine belonging to the family Symplocaceae—has been utilized for the treatment of diabetes. Ethanol extract of *S. cochinchinensis* was reported to down-regulate lipogenesis and enhance insulin sensitivity of a rat model fed with a high fructose and saturated fat diet. Hepatic gene expression and protein profiles were analyzed after administration of the ethanol extract. The results indicated that *S. cochinchinensis* ethanol extract decreased the expression of SCD-1, SREBP-1c, and FAS to modulate lipid accumulation and attenuate hepatic insulin resistance [25]. Another traditional herbal medicine for obesity and diabetes—the root of *Salacia oblonga*, belonging to the family Celastraceae—exhibited a regulation effect on fructose-induced fatty liver in rats. The aqueous-ethanolic extract of *S. oblonga* root diminished lipid steatosis by reducing excess triglyceride accumulation and preventing the increased hepatocellular vacuolization. The fructose-stimulated overexpression of relevant genes was suppressed, such as SREBP-1c, FAS, ACC-1 and SCD-1 mRNA, suggesting that the modulation of the extract is mediated by hepatic SREBP-1c [26]. In addition, the lipid-lowering effect of green tea extract is also partly mediated by its inhibition of liver SREBP-1c and the responsive genes involving FAS and SCD-1 in fructose-fed rats [27].

Besides, it has been pointed out that compounds isolated from herbal medicines show certain bioactivity in controlling hepatic lipogenesis. Silymarin—a flavonolignan isolated from *Silybum marianum*—has emerged as a potential hepatoprotective agent, and was also effective in reducing de novo hepatic lipogenesis. Silymarin treatment significantly depressed the upregulation of SREBP-1c, LXRβ, and FAS genes in the liver of rat model induced by high fructose diet, associated with recovery in insulin sensitivity [28]. Curcumin—a phenolic compound isolated from *Curcuma longa*—has obvious antioxidant and anti-inflammatory properties, which can also prevent high-fructose-induced hyperlipidemia and hepatic steatosis. Administration of curcumin not only obviously lowered triglyceride content and decreased the hepatic protein expression of LXRα and SREBP-1c, but also suppressed the expression of lipogenic enzymes, including ATP-citrate lyase, ACC, and FAS in rats treated with a high-fructose diet [29]. Oleanolic acid—a pentacyclic triterpenoid compound widely distributed in various plants—is clinically applied for hepatoprotective effect in China. This compound showed an inhibitory effect on triglyceride accumulation in fructose-induced rats. The modulation of oleanolic acid was mediated by down-regulating the mRNA expression of SREBP-1c and its nuclear protein expression, which is responsible for de novo lipid synthesis [30]. The anti-steatotic effect of several natural compounds may occur independently of the hepatic signals associated with de novo fatty acid synthesis. For example, mangiferin—a xanthone glucoside derived from *Mangifera indica* and root of *Anemarrhena asphodeloides*—diminished fatty liver in rats treated with fructose by inhibiting hepatic diacylglycerol acyltransferase-2 that catalyzes the final step in triglyceride biosynthesis to reduce the accumulation of triglyceride, instead of SREBP-1c, FAS, ACC-1, and SCD-1 [31]. Genistein [32] and β-conglycinin [33] are also reported to have a regulation effect on fructose-induced hepatic lipogenesis.

### 3.2. Repair of Mitochondrial Dysfunction

An excessive intake of fructose gradually leads to an excess of malonyl-CoA, inhibiting hepatic lipid β-oxidation. The oxidative stress caused by lack of antioxidant capability can be diminished by some natural products targeting leptin, PPARα, and Nrf2.

Aqueous seed extract of *Hunteria umbellata* (belonging to the family Apocynaceae) significantly ameliorated the alterations of blood glucose, insulin, leptin, cholesterol, and triglycerides in high-fructose diet-induced metabolic syndrome rats. Moreover, the extract increased the activities of superoxide dismutase, catalase, glutathione peroxidase, glutathione reductase, glucose 6-phosphate dehydrogenase, and glutathione levels [34]. Polyphenols-enriched extracts from hawthorn fruit peels and fleshes (belonging to Rosaceae) were found to mitigate liver oxidative stress induced by high-fructose diet in mice. Administration of the extracts elevated antioxidant enzyme activities and up-regulated PPARα expression, while down regulating Nrf-2 and antioxidant response element expression to modulate hepatic disorders [35]. The same function as hawthorn, polyphenols-enriched extracts from *Camellia sinensis* (belonging to Theaceae) also modulated lipid homeostasis based on the upregulation of PPARα expression. The administration of the extract ameliorated the fructose-induced hypertriglyceridemia and the insulin-resistance in a fructose-induced hamster model [36].

Oxymatrine—a monosomic alkaloid isolated from *Sophora flavescens*—was found to decrease the liver lipid accumulation by histopathological detection in rats fed with high fructose diet (35% fructose, 35% starch, 10% fat, and 20% protein by energy) for 8 weeks. The research of its activity and mechanism revealed that oxymatrine decreased FAS activity and increased the carnitine palmitoyl transferase 1α (CPT 1α) activity. Additionally, oxymatrine treatment down-regulated the mRNA expression of SREBP-1, FAS, and ACC, and up-regulated the mRNA expression of PPARα, CPT 1α and acyl-CoA oxidase, resulting in the regulation of hepatic lipid metabolism [37]. Betaine, another alkaloid from common edible plants, has been proved effective in treating NAFLD in fructose-induced rat models by up-regulating hepatic expression of LXRα and PPARα with attenuation of the changes of their target genes, including SREBP-1c, ACCα, SCD-1, FAS, CPT I, and CPT II to alleviate hepatic lipid accumulation and fatty acid induced oxidation stress [38]. Thymoquinone—a bioactive benzoquinone isolated from *Nigella sativa* seed—was reported to ameliorate high fructose diet induced depletion of superoxide dismutase and prevent downregulation in hepatic mRNA of PPARα in high fructose diet induced rats to prevent metabolic syndrome [39]. Proanthocyanidin—a polyphenol isolated from grape seed—was reported to increase PPARα more effectively compared to metformin in high-fat-fructose-diet-induced hyperlipidemic rats to promote insulin action [40]. Curcumin was investigated to be useful in the modulation of oxidative stress status and inflammation cascades in rats on high fructose diets, by regulating the serum level of glucose, insulin, leptin, cholesterol, triglycerides, and the expression of NF-κB in hepatocytes [41].

In addition to natural plants, supplementation with dietary *n*-3 fatty acids from fish oil was capable of improving hepatic lipid metabolic response in rats treated with a high-fructose diet as well. The study showed an increase effect on hepatic PPARα gene expression and a decrease effect on gene expression of carbohydrate responsive element binding protein and FAS in rats fed fish oil-rich diets [42].

### 3.3. Inhibition of Inflammatory Pathways

Suffering from fructose-induced oxidation stress—an excess accumulation of intracellular ROS—will lead to triggering inflammation. Excessive intracellular ROS affects the NF-κB pathway via insulin receptor. Several natural products can inhibit the activation of the NF-κB pathway associated with JNK, inflammatory factors like TNF-α and interleukin-6 (IL-6), and insulin receptor substrate-1 (IRS-1) in hepatic inflammation.

Extracts from grape pomace contain relatively high amounts of polyphenols and dietary fiber. Supplementation with grape pomace and its extract altered high-fat-fructose diet-induced activation of JNK in Wistar rats, resulting in a recovery of insulin signaling cascade observed in liver tissue [43]. Extracts from the root of *Withania somnifera* (belonging to Solanaceae) have been confirmed to have anti-inflammatory, antitumor, antioxidant, immunomodulatory, and antistress activities. The latest study suggests that *W. somnifera* normalizes fructose-induced hyperglycemia in rats by reducing the increases of blood glucose, insulin, homeostasis model assessment for insulin resistance, IL-6 and TNF-α, thus alleviating inflammation and improving insulin sensitivity [44].

Isoorientin—a flavonoid isolated from several edible plants—remarkably ameliorated inflammation to inhibit hyperlipidaemia and liver injury by enhancing antioxidant enzyme activities and inhibiting inflammatory cytokine (TNF-α, IL-1, IL-6) secretion in high-fructose-induced obese mice [45]. Curcumin was also reported to attenuate insulin resistance through its anti-inflammatory and antioxidant effects. The administration of curcumin lowered expressions of TNF-α, C reactive protein, cyclo-oxygenase 2, and protein kinase C (PKC)θ, and prevented the activation NF-κB by preventing the degradation of inhibitor of nuclear factor κB (IκB)α in high fructose fed male Wistar rats [46]. (−)-Epicatechin—a flavanol abundant in many dietary plants—downregulated negative regulators such as PKC, IκB kinase, JNK, and protein tyrosine phosphatase 1B in the liver of high fructose-fed rats to mitigate fructose-induced metabolic disorders [47]. Geraniol—a monoterpene alcohol isolated from the essential oil of rose and lemon—prevented the increase of IL-1β and TNF-α in serum, as well as the increase of nitric oxide in liver, modulating fructose-induced inflammation and oxidative stress [48]. Astaxanthin—a xanthophyll carotenoid isolated from *Haemococcus pluvialis*—was reported to inhibit the phosphorylation of JNK1 and IκB-β, production of ROS, and nuclear translocation of NF-κB p65 in liver to alleviate inflammation in high fructose-fat diet-fed mice [49].

### 3.4. Improvement of Insulin Resistance

Liver is a major target tissue of insulin action. Over consumption of fructose stimulates hepatic lipogenesis, mitochondrial dysfunction and inflammation leading to deficiency of insulin sensitivity. Kinds of natural products mitigate insulin resistance through activating PI3K/Akt and AMPK pathways.

Flavonoids extracted from *Lomatogonium rotatum* (belonging to Gentianaceae) enhanced threonine-172 phosphorylation of AMPK and downregulated FAS mRNA expression and leptin levels in liver of high-fructose fed rats. The flavonoids may reduce lipid levels and prevent obesity by stimulating AMPK in hepatocytes [50]. Polysaccharides extracted from *Pleurotus eryngii* (belonging to Pleurotaceae) down-regulated fasting serum glucose and insulin concentrations by enhancing antioxidant activity in the liver of mice fed with fructose diet, with an amelioration of hepatic insulin resistance [51]. Terpenoids extracted from *Liriope platyphylla* (belonging to Liliaceae) altered the expression of Glut1, Glut3, and key proteins in the insulin signaling pathway in the liver of ICR mice treated with high fructose diets to mitigate insulin resistance. The hepatic expression level of Glut3 was down-regulated and Glut1 was up-regulated in response to the phosphorylation of the p38 protein mitogen-activated protein kinase (MAPK) and PI3-K/Akt signaling pathways [52].

Ferulic acid—a phenolic compound isolated from various fruits, vegetables, and some herbal medicines—reduced Glut2 expression by impairing the interaction between Glut2 gene promoter and transcription factors, including SREBP-1c, hepatocyte nuclear factor 1α (HNF1α), and HNF3β in fructose-induced type-2 diabetic rats to improve insulin sensitivity [53]. Resveratrol—a phenol compound isolated from many edible plants—reversed the hepatic triglyceride content in rats induced by high fructose corn syrup by promoting IRS, endothelial nitric oxide synthase, and sirtuin 1, and suppressing FAS and SREBP-1c expression to improve hepatic insulin resistance [54]. This activity was also reported to be partly related to the mRNA expressions of IL-6, IL-10, and IL-18, as well as inducible nitric oxide synthase, Nrf2, and PI3K [55]. In addition, genistein—an isoflavone of soybean used as a kind of phytoestrogen—improved the insulin-stimulated tyrosine phosphorylation of insulin receptor-β and IRS-1, phosphorylation of PI3K and Akt Ser473, and phosphorylation of AMPK Thr172. Significant decrease in IRS-1 serine phosphorylation and S6 kinase-1 Thr389 phosphorylation were observed in high-fat–high-fructose diet-induced mice to attenuate insulin signaling [56]. Astaxanthin—a xanthophyll carotenoid isolated from *Haemococcus pluvialis*—was also reported to alleviate insulin resistance by modulating metabolic enzymes. Liver tissue from astaxanthin-treated mice fed with high-fat–high-fructose diet showed an increase effect on tyrosine phosphorylation and a decrease effect on serine phosphorylation. Astaxanthin increased the association between IRS 1/2 and PI3K and serine phosphorylation of Akt, and decreased fructose-induced serine kinases (JNK-1 and extracellular signal-regulated kinase-1), suggesting that the treatment promotes the IRS-PI3K-Akt pathway of insulin signaling by decreasing serine phosphorylation of IRS proteins [57].

## 4. Conclusions

This paper provided an overview of the current state of knowledge regarding the pathogenesis of fructose-induced NAFLD, and summarized natural products in the literature over the past years with a regulation effect on high fructose-induced NAFLD animal models. Interestingly, these active compounds exert their effects in multi-target manners. For example, phenolics mainly ameliorate NAFLD by affecting the level of SREBP-1c, FAS, SCD, ACC, PPARα, Nrf2, JNK, Glut2, IRS, and PI3K. Curcumin was reported to be effective in hepatic lipogenesis, mitochondrial dysfunction, inflammation, and insulin resistance in high fructose diet animal models. These natural products could provide new starting points in NAFLD new drug research and discovery.

## 5. Prospect

Clinical treatments of NAFLD chiefly includes surgical treatment and drug intervention. Bariatric surgery is one of the treatment options for NAFLD to regulate hepatic glucose and lipid metabolism [58], improve glucose, lipid profiles [59], and insulin resistance [60]. However, as with any major surgery, the procedure does carry some risks. Drug intervention is another method in NAFLD therapy. Metformin (dimethylbiguanide)—derived from active compound of *Galega officinalis* (Fabaceae) [61]—is an AMPK-activating molecule that has been used in the treatment of type-2 diabetes for more than 50 years [24]. It can decrease the levels of serum aminotransferases in patients with histologically confirmed NAFLD during the first 3 months of treatment [62]. However, the low response rate cannot meet the clinical requirements.

Obviously, it is urgent to develop therapeutic and preventive strategies for NAFLD. Natural products could provide new starting points in new NAFLD drug research and discovery. More and more researchers are paying attention to natural products to solve the emergent condition on NAFLD. According to studies over the latest years, effective natural products on fructose-induced NAFLD mainly focus on phenolics, flavonoids, alkaloids, and terpenoids. These compounds are absolutely different in chemical structures, but their targets may be similar to some degree. Therefore, it is difficult to ascertain how they could regulate the same target in NAFLD. Quantitative structure–activity relationship research methods can be applied in this field to provide scientific evidence. Although the activity of natural products is not stronger than that of synthetic compounds, it also supplies a potential source to develop better new drugs or dietary supplements for NAFLD.

## Abbreviations

ACC	Acetyl-CoA carboxylase
ACLY	ATP-citrate lyase
ACS	Acyl-CoA synthase
AMPK	Adenosine 5′-monophosphate (AMP)-activated protein kinase
ATGL	Adipose triglyceride lipase
CPT-1	Carnitine palmitoyl transferase 1
DGAT	Diacylglycerol acyltransferase
eNOS	Endothelial nitric oxide synthase
FAS	Fatty acid synthase
FFA	Free fatty acid
Fru	Fructose
Fru-1-P	Fructose-1-phophate
Glut5	Glucose transporter 5
GPAT	Glycerol-3-phosphate acyltransferase
HNF1α	Hepatocyte nuclear factor 1α
HSL	Hormone-sensitive lipase
IKK	Inhibitor of nuclear factor κB (IκB) kinase
IL-6	Interleukin-6
iNOS	Inducible nitric oxide synthase
IR	Insulin receptor
IRS-1	Insulin receptor substrate-1
JNK	c-Jun amino terminal kinase
LKB1	Liver kinase B1
LXRα	Liver X receptor α
MAPK	Mitogen-activated protein kinase
MCD	Malonyl-CoA decarboxylase
mTOR	Mammalian target of rapamycin
NAFLD	Nonalcoholic fatty liver disease
NF-κB	Nuclear factor kappa B
Nrf2	Nuclear factor-erythroid 2-related factor 2
PI3K	Phosphoinositide 3-kinase
PKA	Protein kinase A
PKC	Protein kinase C
PPARα	Peroxisome proliferator–activated receptor α
PPARγ	Peroxisome proliferator activated receptor γ
ROS	Reactive oxygen species
SCD	Stearoyl-CoA desaturase
SREBP-1c	Sterol regulatory element binding protein 1c
TAG	Triglyceride
TNF-α	Tumor necrosis factor α
VLDL	Very low density lipoprotein
